# Centromere identity from the DNA point of view

**DOI:** 10.1007/s00412-014-0462-0

**Published:** 2014-04-25

**Authors:** Miroslav Plohl, Nevenka Meštrović, Brankica Mravinac

**Affiliations:** Division of Molecular Biology, Ruđer Bošković Institute, Bijenička 54, 10002 Zagreb, Croatia

**Keywords:** Centromere, Satellite DNA, Transposable elements, Transcription

## Abstract

The centromere is a chromosomal locus responsible for the faithful segregation of genetic material during cell division. It has become evident that centromeres can be established literally on any DNA sequence, and the possible synergy between DNA sequences and the most prominent centromere identifiers, protein components, and epigenetic marks remains uncertain. However, some evolutionary preferences seem to exist, and long-term established centromeres are frequently formed on long arrays of satellite DNAs and/or transposable elements. Recent progress in understanding functional centromere sequences is based largely on the high-resolution DNA mapping of sequences that interact with the centromere-specific histone H3 variant, the most reliable marker of active centromeres. In addition, sequence assembly and mapping of large repetitive centromeric regions, as well as comparative genome analyses offer insight into their complex organization and evolution. The rapidly advancing field of transcription in centromere regions highlights the functional importance of centromeric transcripts. Here, we comprehensively review the current state of knowledge on the composition and functionality of DNA sequences underlying active centromeres and discuss their contribution to the functioning of different centromere types in higher eukaryotes.

## Introduction

An essential function of genetic material in any living organism is its faithful segregation, the role which is in eukaryotes determined by the centromere. The centromere includes the core or functional centromere domain, a specialized locus at which microtubules attach to the complex multiprotein structure of the kinetochore in order to segregate chromosomes in mitosis and meiosis. The core centromere domain is surrounded by large blocks of pericentromeric heterochromatin (also called the pericentromere), primary sites of sister chromatid cohesion. Centromere functionality is vital for all eukaryotic organisms. In addition to understanding its role as a biological structure, studying the centromere is also highly relevant from a biomedical point of view, because abnormalities in centromeric function are often lethal or associated with various congenital and acquired diseases, such as cancer, infertility, and birth disorders (reviewed in Thompson et al. 2010).

Centromeres are considered to be shaped by both genomic and epigenetic mechanisms, but the synergy between DNA sequences, protein components, and epigenetic marks is still not well understood. In the absence of a universal DNA sequence, species-specific histone H3 variant CENH3 (CENP-A in mammals, CID in *Drosophila melanogaster*, Cse4 in *Saccharomyces cerevisiae*) is the most prominent protein identifier of centromere function. Related forms of this protein have been detected in all studied active centromeres of single-cell and multicellular eukaryotes (Black and Bassett 2008; Malik and Henikoff 2009). CENH3 replaces the canonical histone H3 in such a way that arrays of CENH3-based nucleosomes alternate with those containing canonical H3 (Blower et al. 2002; Sullivan and Karpen 2004). In humans and flies, canonical H3 is in turn epigenetically modified in the centromere, by dimethylation at lysine 4 (H3K4me2), and thus distinctive from the histone H3 in adjacent pericentromeric heterochromatin, which is marked by methylation at lysine 9 (H3K9me). These differences qualify centromeric chromatin as a unique chromatin type centrochromatin (Sullivan and Karpen 2004).

In the budding yeast *S. cerevisiae*, centromere function depends on a short, about 100 bp long DNA sequence motif. These centromeres are referred to as simple or point centromeres (Hyman and Sorger 1995). In all other eukaryotes, centromeres are founded on repetitive DNA arrays of several hundred kilobase, commonly known as complex or regional centromeres (Pluta et al. 1995). A single centromere is normally formed on each chromosome in a locus which is on the cytogenetical level recognized as a primary constriction of the monocentric chromosome. However, there are exceptions, and some organisms have holocentric chromosomes that lack a primary constriction and comprise of a centromere dispersed in many subdomains along the entire chromosome length (Dernburg 2001).

Mostly, due to limitations in sequencing and assembly of long arrays of nearly-identical repeats, our knowledge on the long-range functional organization of centromeric DNA is rather limited, and centromeres still represent the last frontiers in genome assemblies and sequence annotations (Hayden and Willard 2012). Here, we review the rapidly progressing field of functional centromere genomics. We present data relating DNA sequences and their functional interactions in different centromere types of higher eukaryotes, and point to the significance of transcriptional potential of centromeric sequences.

## Repetitive DNA sequences are the most common centromere components

Two classes of highly abundant repetitive sequences, satellite DNAs (satDNAs) and transposable elements (TEs), represent major DNA components of many centromeric regions. Both groups of sequences are extremely divergent, and understanding the mechanisms of their accumulation, diversification, protein-binding capacity, and linear distribution is essential for a complete picture of centromere genomics, both from a structural and functional perspective. Characteristics of functional DNA sequences and other abundant DNAs contributing to centromere region of the most common model organisms of higher eukaryotes are presented in Table 1.

**Table 1:** Centromere DNA features in higher eukaryote model organisms

Centromere type according to functional DNA sequence	Species/common name	Characteristics of functional DNA sequence(s)	Other abundant DNAs contributing to centromere region	References
Satellite DNA	*Homo sapiens*/human	alpha-satDNA [171 bp] in all centromeres; chromosome-specific subfamilies; higher-order organization	Monomeric forms of alpha-satDNA, diverse non-alphoid satDNAs (gamma, beta, Sat I, II, III) and LINE elements in pericentromeric regions	Willard and Waye 1987; Waye and Willard 1989; Rudd and Willard 2004; Rudd et al. 2006; Sullivan et al. 2011; Schueler et al. 2001
	*Drosophila melanogaster*/fruit fly	*Dp1187* centromere: AATAT and AAGAG satDNAs	*Dp1187* centromere: LTR retrotransposons (HMS Beagle, 412, and Bel), non-LTR (LINE-like) retroposon (*F*) and 359-bp satDNA	Sun et al. 1997, 2003
	*Mus musculus*/house mouse	Minor satDNA [120 bp]: homogenous family in all centromeres	satDNAs: MS3 [150 bp] in centromeric core, Major satDNA [234 bp] and MS4 [300 bp] in pericentromeric regions	Guenatri et al. 2004; Kuznetsova et al. 2006
	*Arabidopsis thaliana*/thale cress	pAL1 satDNA [180 bp]: homogenous family in all centromeres	LTR-retrotransposon (Athila) in centromere core, multiple families of LTR retrotransposons and 5SrRNA in pericentromeric regions	Nagaki et al. 2003; Kumekawa et al. 2000
	*Pisum sativum*/pea	13 distinct satDNAs families [50–2,094 bp] localized in various combinations in different centromeres	satDNAs: TR2, TR3, and TR5 in pericentromeric regions	Neumann et al. 2012
Satellite DNAs and retrotransposons	*Oryza sativa*/rice	CentO satDNA [155 bp] and CRR retrotransposon in all centromeres	Different retrotransposon families belonging to Ty3/gypsy–class	Dong et al. 1998; Cheng et al. 2002
	*Zea mays*/maize	Retrotransposons CRM1 and CRM2 and CenC satDNA [156 bp] in all centromeres	Retrotransposons CRM3 and CRM4	Zhong et al. 2002; Wolfgruber et al. 2009
Retrotransposons	*Triticum* spp*.*/wheat	CRW, Quinta and Weg retrotransposon families	Different CRW retrotransposons families (Ty3/gypsy–class)	Li et al. 2013
Repeats and non-repeats	*Equus caballus*/horse	satDNAs: different ECA families [221–475 bp], 37cen [221 bp], and 2PI [23 bp] localized in various combinations; repeat-free ch11 centromere	NA	Piras et al. 2010; Alkan et al. 2011
	*Gallus gallus*/chicken	Chromosome specific satDNAs [1.8–3.2 kb] in centromeres of eight macrochromosomes, CNM satDNA [42 bp] in some microchromosomes and in ch6 and ch9; repeat-free ch5, ch27, chZ centromeres	NA	Shang et al. 2010
	*Solanum tuberosum*/potato	Six chromosome specific satDNAs [979 bp to 5.4 kb]; repeat-free ch4, ch6, ch10, ch11, and ch12 centromeres	NA	Gong et al. 2012

SatDNAs are a class of diverse tandemly repeated DNA sequences that comprise long arrays localized in a tightly packed heterochromatin. Features of satDNA sequences in centromeric regions have already been reviewed in detail (Plohl et al. 2008, 2012). A recent comprehensive bioinformatic analysis of centromeric satDNAs in a number of animal and plant species confirmed the rapid evolution of DNA sequences in these areas (Melters et al. 2013). Despite the extreme diversity of satDNA sequences, some sequence segments can be shared among heterologous repeats. The best known example is the conserved 17 bp long sequence motif, the CENP-B box, which is specific for alpha-satDNA in humans (Ohzeki et al. 2002), as well as in various subclasses of alphoid repeats in mammalian species (Alkan et al. 2011). This motif is a binding site for the protein CENP-B, which probably facilitates kinetochore formation (Masumoto et al. 2004), but might also play a role in rearrangements of satDNA sequences (Kipling and Warburton 1997). The presence of CENP-B box-like motifs in unrelated satDNAs of some distant invertebrates and plants suggests its potential functional relevance in non-mammalian organisms (Mravinac et al. 2005; Canapa et al. 2000; Meštrović et al. 2013; Gindullis et al. 2001).

SatDNAs evolve according to the principles of concerted evolution. Within the genome, mutations are homogenized among repeats of the satDNA by the mechanisms of non-reciprocal sequence transfer, such as unequal crossover, gene conversion, rolling circle replication, and transposition-related mechanisms (Dover 1986). Although the centromere was traditionally treated as a region of suppressed recombination, unequal crossing-over and gene conversion have been identified as the most widespread mechanism involved in satDNA dynamics (Mahtani and Willard 1998; Smith 1976; Talbert and Henikoff 2010). Nevertheless, recent studies on primates and plants postulated mechanism of segmental duplication as an important evolutionary force in the massive amplifications of satDNA arrays and long range rearrangements of (peri)centromere regions (Horvath et al. 2005; Ma and Jackson 2006). At the population level, satDNAs become fixed as a result of random assortment of genetic material in meiosis. As species diverge, satDNAs accumulate changes as a consequence of mutations and turnover mechanisms in separate lineages generating species-specific satDNA arrays (Dover 1986). However, rapidly accumulating differences in species-specific satDNA profiles can also be accomplished by amplifications/contractions of repeats existing in a so-called library of satDNAs common to related genomes. The hypothesis was originally proposed by Fry and Salser (1977) and experimentally proved by Meštrović et al. (1998). As predicted by the theory of concerted evolution, a small bias in favor of homogenization of a particular set of repeat variants would lead to extreme conservation of satDNAs (Ohta and Dover 1984; Strachan et al. 1985), observed in various organisms, for example, in sturgeons (De la Herran et al. 2001) and beetles (Mravinac et al. 2002). Because of the above mentioned specificities, the scenario of satDNA evolution unifies array homogeneity and long-term sequence stability together with the ability of the satDNA library to act as a reservoir of sequences that allow rapid changes through expansions and contractions of arrays (Plohl et al. 2008).

Nevertheless, it is difficult to understand the rapid evolution of satDNAs in a centromere solely by sequence dynamics of tandem repeats, especially in the light of the centromere structure-function paradox (Eichler 1999). The phenomenon of rapid evolution of centromeric DNA and protein components in spite of conserved centromere function has been referred to as the centromere paradox (Henikoff et al. 2001). In this regard, evolution of CENH3 is subject to positive selection in *Drosophila* (Malik and Henikoff 2001) and *Arabidopsis* (Talbert et al. 2002), and probably in general (Talbert et al. 2004) because of its interactions with changing DNA components. Centromeres are thus not defined only by epigenetic factors but also through interactions between repetitive DNA and protein components, mediated by meiotic drive (Dawe and Henikoff 2006). In other words, rapid evolution of centromere satDNA sequences is possible only assuming coevolution with CENH3 and other DNA-binding proteins.

Because satDNAs are the major DNA components of heterochromatin, differences in their composition can be linked with reproductive isolation and speciation (Bachmann et al. 1989). Differences among individuals in the centromere region accumulate as a consequence of centromere drive, leading to reduced compatibility of homologous chromosomes in hybrids and ultimately to postzygotic isolation, thus triggering speciation (Henikoff et al. 2001). The role of satDNA in reproductive isolation caused by rapid centromere evolution has been recently studied in detail in monkey-flowers (Fishman and Saunders 2008) and *Drosophila* (Ferree and Barbash 2009).

Another repetitive component of importance for centromeric regions are transposable elements (TEs), DNA sequences which can move to new genomic locations and form interspersed repeats if replicated in the process of movement (Kazazian 2004; Tollis and Boissinot 2012). According to the mechanisms of transposition, TEs are categorized as RNA-mediated (retroelements such as long terminal repeat (LTR) and non-LTR-retrotransposons) or DNA-mediated (DNA transposons). In addition to sequence segments coding for their own enzymes and thus being self-sufficient in the process of mobility, enzymes of autonomous elements can trail a large number of various non-autonomous copies.

Among TEs, LTR-retrotransposons in particular accumulate frequently in centromeres and pericentromeres of both plants and animals (e.g., Pimpinelli et al. 1995; Copenhaver et al. 1999; Schueler et al. 2001; Cheng et al. 2002). TEs belonging to the chromovirus clade of Ty3/gypsy LTR-retrotransposons are widely distributed in centromeres of angiosperms. It has been proposed that they are targeted to centromeres by a specific motif located at the C-terminus of their integrase (Neumann et al. 2011). Molecular determinants that need to be recognized by this motif in order to trigger specific integration are probably sequence-independent heterochromatin marks, although their exact nature has not yet been unambiguously identified (Neumann et al. 2011; Tsukahara et al. 2012). In addition to active transposition, centromere-specific retrotransposons can become significantly enriched in centromeric regions as a consequence of multiple rounds of segmental duplication, a process which can also be responsible for massive amplifications of satDNA arrays (Ma and Jackson 2006).

Despite differences in the structure, organization, dynamics, and mechanisms of spread, a growing number of reports link TEs and satDNAs. A whole unit or a segment of a TE can be amplified in tandem, although the direction of transition between the two types of repetitive sequences is not always clear (Macas et al. 2009). For example, a part of the mammalian retrotransposon L1 shares similarity with a segment of the satDNA repeat in whales (Kapitonov et al. 1998). Internal tandem repeats of non-autonomous miniature inverted repeat transposable element (MITE) from the cupped oyster *Crassostrea virginica* resemble satDNAs in several other mollusks (Gaffney et al. 2003). In plants, a hypervariable region of one LTR-retrotransposon was found expanded into tandem repeats of a satDNA in the pea (*Pisum sativum*) genome (Macas et al. 2009). Similarly, *Zea mays* centromeres became enriched in tandem repeats derived from LTRs and untranslated regions of two unrelated centromere-specific retrotransposons, what probably happened in two independent evolutionary events (Sharma et al. 2013).

## Repeat-based centromeres

The majority of eukaryotes studied in terms of centromeric DNA have monocentric chromosomes with large regional centromeres. Functional centromeric domains of these chromosomes are usually inserted into blocks of pericentromeric heterochromatin, a compartment composed of Mb-sized arrays of satDNAs. Arrays are in general much longer than necessary for centromeric function. For instance, functional centromere domains in *Drosophila* comprise only of 15–40 kb, which is comparable to the minimum length of 30–70 kb of alpha-satDNA in a functional centromere of human artificial chromosomes (Okamoto et al. 2007).

**Fig. 1:**
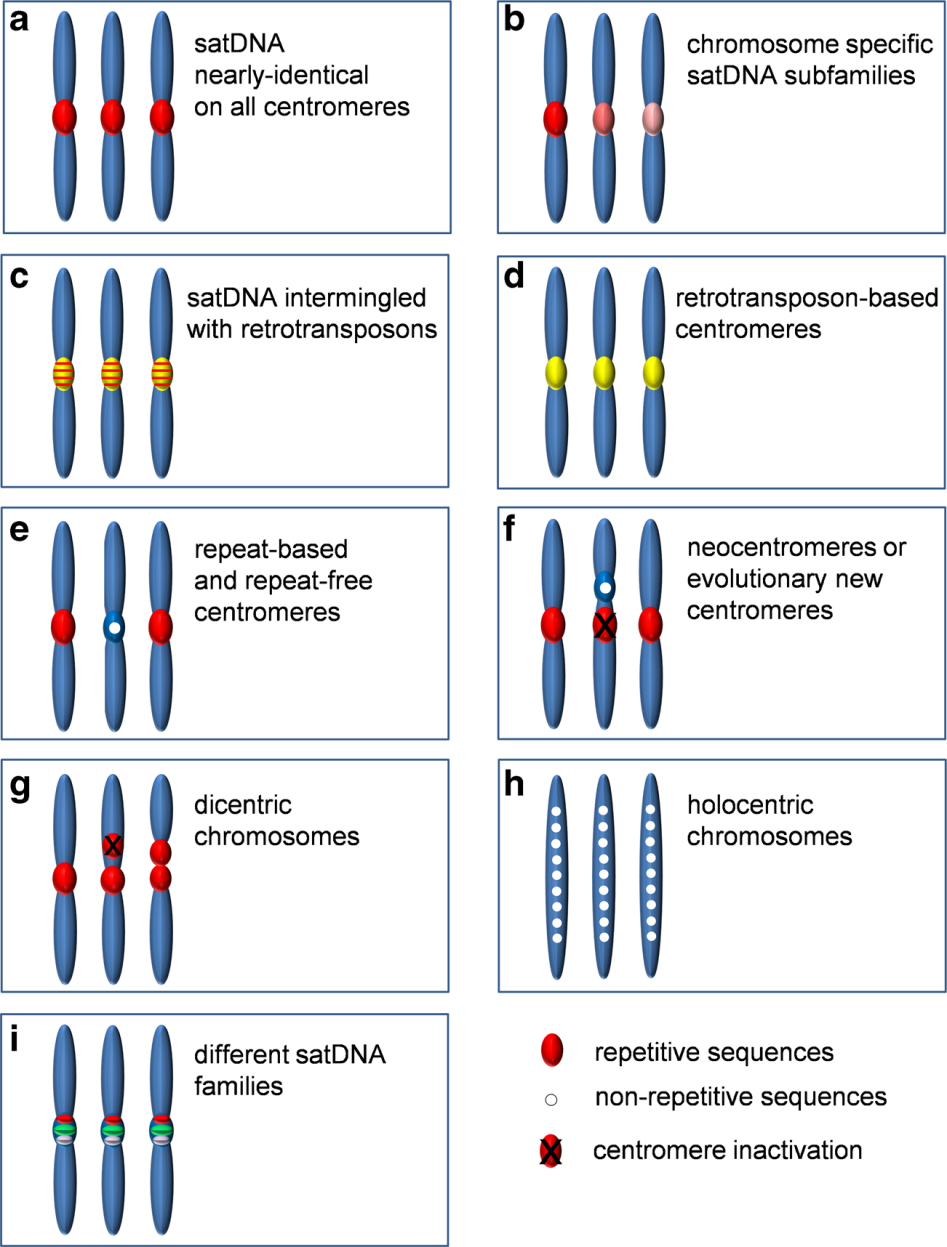
Schematic presentation of functional DNA sequences in different centromere types

Details on the complexity of organizational patterns and contribution of particular sequence types to repeat-based centromeres differ significantly among species (Fig. 1). For example, global sequence characterization of rice centromeric satDNA CentO by next generation high-throughput sequencing and ChIP experiments with CENH3 could not reveal any particular differences between monomers included in the functional centromere and pericentromeric arrays (Macas et al. 2010). A comparable uniform distribution of nearly-identical repeats of species-specific highly-abundant satDNAs (up to 50 % of the genome) in centromeric and pericentromeric heterochromatin of all chromosomes can be anticipated in some beetle species of the order Coleoptera (Palomeque and Lorite 2008). It has been proposed that the lack of chromosome-specific satDNA variants (Fig. 1a) indicates high efficiency of sequence homogenization in the bouquet stage of meiotic prophase, in which all chromosomes of the complement align together (Durajlija Žinić et al. 2000; Mravinac and Plohl 2010). In contrast, well-known examples of satDNAs localizing to pericentromeric and centromeric regions are the mouse major and minor satDNA, respectively, (Guenatri et al. 2004; Kuznetsova et al. 2006).

The distribution of centromeric satDNAs can also be chromosome specific (Fig. 1b). The best studied example is the complex organizational pattern of centromeric sequences in human chromosomes. Two basic types of alpha-satDNA, monomeric and higher-order repeat (HOR), characterize human centromeric regions (Willard and Waye 1987; Rudd and Willard 2004). All regular human centromeres are formed on tandemly repeated HOR units composed of 2 to over 20 diverged 171-bp-long monomers, and HORs are usually chromosome specific (Rudd et al. 2006). However, only a fraction of HOR arrays of human alpha-satDNA underlies active centromeres, while the rest, flanked by monomeric repeats, contributes to pericentromeric heterochromatin (Spence et al. 2002; Lam et al. 2006; Mravinac et al. 2009; Sullivan et al. 2011). Comparably, in the domestic dog, CENP-A chromatin immunoprecipitation (ChIP) experiments suggested monomer sequence subtypes of two related satDNAs as functional centromere sequences (Hayden and Willard 2012). Recent efforts combining genomic and ChIP-obtained data on human alpha-satDNA allowed the possibility for comprehensive functional mapping of centromeric areas and led to a model in which the centromere is defined by sequence features and context-dependent epigenetic interactions (Hayden et al. 2013).

The diversity of DNA sequences localized in functional centromeres and/or pericentromeres has been evidenced not only in terms of different satDNAs and their organizational forms, but also in terms of other sequences’ contribution. Different interspersion patterns of tandemly repeated DNA and TEs are found in many species (Fig. 1c). The centromeric fraction of human HORs is mostly devoid of inserted TEs or other sequences, while pericentromeres are frequently interrupted by unrelated satDNAs (e.g., gamma-satellite and SatIII) and LINE elements (Schueler et al. 2001). Different plants such as maize, rice, and wheat turned out to be valuable models for studying the specificities of centromere DNA sequence organization, particularly because of the presence of substantial portions of centromere-specific retrotransposons. Retrotransposons are extensively intermingled with satDNAs and both sequence types mark functional parts of some plant centromeres (Ma et al. 2007). For instance, functional rice centromeres are characterized by CentO satDNA and the centromere-specific retrotransposon CRR (Cheng et al. 2002). A recent study in the wild rice *Oryza brachyantha* showed that CentO satDNA repeats as well as CRR retrotransposons have completely disappeared and are replaced by a new functional centromeric CentF satDNA in a short evolutionary time (Lee et al. 2005).

Detailed mapping of the repeat content and arrays of complete centromeres in some chromosomes of maize (Wolfgruber et al. 2009) and wheat (Li et al. 2013) revealed species-specific centromeric retrotransposons as predominant CENH3-associated DNA sequences (Fig. 1d). Maize centromeres still contain small amounts of CentC satDNAs, detected as functional centromeric sequences in other maize inbreds (Kato et al. 2004; Wolfgruber et al. 2009) and related to the CentO satDNA in rice (Cheng et al. 2002). Similar replacements of functional centromeric satDNA with retrotransposons occurred in wheat, followed by consecutive introduction of new functional retrotransposons. All these replacements occurred in a very short evolutionary time, <0.5 MY (Li et al. 2013). In principle, older retrotransposons typically lie outside of the functional centromere (Wolfgruber et al. 2009; Li et al. 2013) and can be compared with the distribution of LINE and other TEs in pericentromeres of human chromosomes (Schueler et al. 2001). It has been hypothesized that retrotransposons may accumulate in active centromeres because of favored integration into an epigenetically modified centromere environment, and not because of preferred association with CENH3 nucleosomes (Lamb et al. 2007; Wolfgruber et al. 2009).

Complex organization of centromeric regions is further supported by the presence of protein coding genes or gene candidates in centromeric chromatin of *D. melanogaster* (Smith et al. 2007), rice (Wu et al. 2004; Nagaki et al. 2004), and wheat (Li et al. 2013), although the insertions of this type were not observed in *Arabidopsis* (Hosouchi et al. 2002) and human (Schueler et al. 2001).

## Organisms with both repeat-based and repeat-free centromeres

From the methodological standpoint, due to the abundance of satellite repeats in eukaryotic species, it is understandable that the literature to date mostly describes the cases of centromeric regions rich in repetitive sequences. However, the development of chromatin immunoprecipitation and usage of CENH3 variants as the most reliable markers of active centromeres enabled high-resolution DNA mapping of interacting sequences. Consequently, there are an increasing number of reports documenting the organisms that possess both repeat-based and repeat-free centromeres (Fig. 1e). Horse *Equus caballus* centromeres are enriched for satellite sequences but the functional centromere of chromosome 11 lacks any tandem repeats (Piras et al. 2010). The extended cytogenetic analysis of congeneric species revealed that donkey and two zebra species contain several pairs of chromosomes with satellite-less centromeres (Piras et al. 2010). The chicken genome with 10 pairs of macrochromosomes, 28 pairs of microchromosomes, and Z/W sex chromosomes represents the first avian karyotype with molecular cytogenetic characterization of each chromosome (Masabanda et al. 2004), and thus has been a powerful resource for studying the genetic makeup. Thorough identification of centromeric DNA showed that the majority of chicken centromeres are founded on chromosome-specific satDNA spanning several hundred kilobase of homogeneous repetitive arrays, while centromeres of chromosomes 5, 27, and Z, spanning only ~30 kb, are devoid of tandem repeats (Shang et al. 2010). The presence of the two distinct types of centromeres has also been evidenced in plants. In the potato, *Solanum tuberosum*, no satellite repeats were discovered in centromeres of five pairs of chromosomes, whereas six potato centromeres harbor megabase-sized chromosome-specific satellite repeat arrays (Gong et al. 2012). Similar to chicken, centromeric satellites in potato share partial sequence similarity to different retrotransposon sequences (Gong et al. 2012).

## Neocentromeres and evolutionary new centromeres (ENCs)

Neocentromeres are fully functional centromeres that arise at ectopic DNA loci not previously associated with kinetochore proteins (Fig. 1f). In humans, the majority of neocentromeres evidenced in clinical phenotypes rescue acentric chromosome fragments in cells with severe chromosomal rearrangements (Marshall et al. 2008). As the neocentromeres described to date show notable divergence of underlying DNA sequences and chromosome positions, the sequence attributes that might be favorable to their formation have not yet been established. Most of them are located in gene-poor regions with no apparent association with heterochromatin (Alonso et al. 2010), and although some of them form on repetitive DNA (Hasson et al. 2011), none of them are associated with alpha-satellite DNA. In addition to human cells, neocentromere formation and function have also been studied in different model organisms such as *D. melanogaster*, *Schizosaccharomyces pombe*, *Candida albicans*, and several plant species (reviewed in Burrack and Berman 2012).

Evolutionary new centromeres (ENCs), also known as repositioned centromeres, are centromeres that moved to a new position along a single chromosome without any observable chromosomal rearrangements or phenotypic consequences. Once repositioned, ENCs are transmitted through generations and become fixed in the population. Since they can be identified exclusively by comparing the ancestral and derived position of a specific centromere, systematic karyotype analyses of related organisms are crucial. So far, the best studied model group is primates and it has been proved that nine macaque chromosomes possess ENCs (Ventura et al. 2007), whilst six human centromeres are evolutionarily new (reviewed in Rocchi et al. 2012). ENCs have also been revealed in other mammals (e.g., Carbone et al. 2006; Rocchi et al. 2012), birds (Kasai et al. 2003), and plants (Han et al. 2009). Although they arise in anonymous sequences, ENCs gradually incorporate repetitive arrays. In macaque, all the nine ENCs over time accumulated large arrays of alpha-satDNA becoming indistinguishable from other macaque centromeres. At the same time, the inactivated centromeres completely lost their satellite arrays (Ventura et al. 2007). Similarly, centromere repositioning in cucurbit species was accompanied by the gain of centromeric satDNA repeats in ENCs and the loss of pericentromeric heterochromatin in inactivated centromeres (Han et al. 2009).

What can be learned from neocentromere and ENC phenomena is that a centromere potentially can be seeded in any unique sequence, albeit the repetitive DNA setup provides a preferred chromatin environment for centromere maintenance. The hypothesis that repeat-free centromeres represent a primordial form is in accordance with the occurrence of neocentromeres and their maturation into repeat-based centromeres by the accumulation of satellites and retrotransposons (Kalitsis and Choo 2012).

## Dicentric chromosomes

Each chromosome normally possesses a single centromere, though genome rearrangements can generate chromosomes with two centromeres (Fig. 1g). In general, dicentric chromosomes are inherently very unstable because of anaphase bridge formation resulting in broken or rearranged chromosomes. Nevertheless, in some cases, dicentric chromosomes are stabilized due to inactivation of one of the two centromeres, which allows the structural dicentric to act as a functional monocentric during cell divisions. The exact mechanism of centromere inactivation has not been completely elucidated; however, studies of naturally occurring and engineered dicentrics in different organisms predominantly indicate epigenetic changes. In the fission yeast, *S. pombe*, 99 % of the cells harboring an artificial dicentric chromosome died, but in 70 % of the survivors, one of the centromeres was functionally silenced by the loss of Cnp1 (the yeast CENH3 homolog), depletion of euchromatic histone modifications H3K9ac and H3K14ac, and by becoming enriched for the heterochromatic H3K9me2 mark without associated alterations in the DNA sequence (Sato et al. 2012). Epigenetic centromere inactivation has also been documented in maize dicentric B chromosomes. Without changing the sequence of underlying DNA, one of the B chromosome centromeres becomes nonfunctional by histone CENH3 depletion (Han et al. 2006) and increasing methylation of the underlying DNA (Koo et al. 2011). A structural tricentric chromosome in wheat acts like a functional monocentric by keeping active the large centromere, while at the same time both of the small centromeres, enriched for heterochromatic histone modifications H3K27me2 and H3K27me3, are inactivated (Zhang et al. 2010). Dicentric chromosomes in humans can be quite stable, and it has been known for two decades that some human dicentric chromosomes also stay functional dicentrics through multiple cell divisions (Sullivan and Willard 1998). Stimpson et al. (2010) recently showed that the human dicentrics, being functionally monocentric, undergo centromere inactivation through different processes: (1) by epigenetic mechanisms or (2) by size reduction of the alpha-satDNA array associated with CENP-A. Human chromosome HSA17, characterized by the two alpha-satellite arrays D17Z1 and D17Z1-B, is an example of a regular human chromosome structurally arranged as a dicentric that behaves as a functional monocentric. Its functional centromere is predominantly linked to the D17Z1 array (Maloney et al. 2012). However, in vitro and in vivo studies proved that the HSA17 functional centromere can also assemble at D17Z1-B, and its location is inherited through multigenerational families. The structural differences in the D17Z1 and D17Z1-B HOR arrays imply genomic factors that, together with epigenetic mechanisms, influence centromere specification in humans (Maloney et al. 2012). In other words, the analyses of natural and engineered dicentric chromosomes indicate that epigenetic plasticity, but also subtle genetic features of centromere-competent DNA sequences, plays an important role in defining centromere identity.

## Holocentric centromeres

In contrast to monocentric, holocentric chromosomes have a long kinetochore plate with spindle fibers attached along the entire chromosome length (Dernburg 2001) (Fig. 1h). Based on cytological studies, it has been shown that holocentric chromosomes are scattered among plant and animal kingdoms arising at least 13 independent times during evolution (Mola and Papeschi 2006). A more precise understanding of centromeric function in holocentric species, based on immunodetection of CENH3 homologs, has been intensively analyzed only in the nematode, *Caenorhabditis elegans*, and a few other species. In spite of polyphyletic origin, immunodetection of the corresponding CENH3 proteins in mitotic chromosomes of *C. elegans* (Buchwitz et al. 1999) and the plant *Luzula* (Nagaki et al. 2005; Heckmann et al. 2011) shows common structural features in the form of dispersed CENH3 distribution during interphase and prophase. In both species, diffuse centromeres are distributed along each chromatid except in the telomeric regions (Heckmann et al. 2011). Data on the DNA sequences underlying holocentric centromeres are generally lacking. Nevertheless, a recent study of animal and plant species shows that the genomic content of tandem repeats in holocentric species differs greatly (Melters et al. 2013). The *C. elegans* genome contains only a few tandem repeats (Hillier et al. 2007). ChIP analysis shows that even ~50 % of this genome is associated with CENH3, but association loci are not correlated with repeat density (Gassmann et al. 2012). In contrast, comprehensive characterization of holocentric *Luzula elegans* shows that 61 % of its genome is built of highly repetitive DNAs, including over 30 highly divergent satellite families, while 33 % of the genome comprises Ty1/copia LTR retrotransposons of the Angela clade (Heckmann et al. 2013). Although retrotransposons in *L. elegans* are uniformly distributed along the chromosomes, they are not centromere-associated. Similarly, different satDNAs are present as blocks preferentially accumulated on chromosome ends which are declared as non-centromeric regions. However, a portion of centromere domains in the related holocentric species *Luzula nivea* is composed of scattered clusters of satellite LCS1 which display significant similarity to the major centromeric satellite of monocentric chromosomes of some *Oryza* species (Haizel et al. 2005). These data suggest that satDNA can be an important centromere determinant in this holocentric species. In support of this, a study of novel meta-polycentric chromosomes in the pea *P. sativum*, which represents the first example of an intermediate between monocentric and holocentric centromeres, demonstrates that all functional centromere domains in the pea are tightly associated with clusters of 13 distinct satDNA families and with one family of retrotransposons (Neumann et al. 2012). The pea centromeres have from three to five explicit CENH3-containing regions composed of different families of satDNAs (Fig. 1i).

## Transcription of centromeric sequences

The non-coding nature of repetitive sequences in centromeres and pericentromeres led to the opinion that centromeres are transcriptionally inactive. However, new evidences show that small-interfering RNAs (siRNAs) transcribed from pericentromeric tandem repeats in *S. pombe* modify the heterochromatin. In brief, transcription of pericentromeric sequences in the form of double stranded RNAs and their processing into siRNAs by the ribonuclease Dicer proved to be crucial in heterochromatin assembly and transcriptional silencing (Volpe et al. 2002). Impairment of the RNA interference (RNAi) pathway resulted in severe chromosome segregation defects in *S. pombe* (Hall et al. 2003). Subsequent studies on higher eukaryotic species showed a link between the RNAi machinery and heterochromatin-mediated transcriptional silencing in plants (Zilberman et al. 2003), flies (*Drosophila*; Pal-Bhadra et al. 2004), worms (*C. elegans*; Grishok et al. 2000), and mammals (Fukagawa et al. 2004). However, the ultimate impact of RNAi on heterochromatin assembly and chromosome segregation is less straightforward suggesting different mechanisms of the RNAi pathway in complex genomes (Chan and Wong 2012). In hybrid chicken cells carrying a human chromosome, loss of Dicer led to defects in centromere heterochromatin and chromosome segregation, pointing out the importance of siRNA for heterochromatin assembly (Fukagawa et al. 2004). Similarly to chicken cells, Dicer deficiency in mouse embryonic stem (ES) cells caused accumulation of pericentric satellite transcripts, but there are still controversies related to the impact of the RNAi machinery on mammalian centromere assembly (Kanellopoulou et al. 2005; Murchison et al. 2005). Kanellopoulou et al. (2005) reported loss of DNA methylation and of histone H3 modification H3K9me3 at the pericentromeric regions of Dicer-deficient ES cells and suggested that Dicer participates in the maintenance of centromeric heterochromatin structure. In contrast, Murchison et al. (2005) concluded that the RNAi pathway is not essential for the regulation of heterochromatin assembly in mouse ES cells because in their experimental system Dicer loss had no significant effect on cytosine methylation nor changed H3K9me3 status at the centromere. More recent work on *S. pombe* suggests that the observed defects may be indirectly related to exosome RNA machinery (a multiprotein complex capable of degrading various RNA types), which acts in parallel with RNAi and promotes heterochromatin formation (Reyes-Turcu et al. 2011).

In addition, a great progress has also been made in determining non-siRNAs transcripts in the centromere of higher eukaryotes. The data suggest transcriptional competence of the entire centromere (both the centromere core and the pericentromere) and heterogenous transcripts appear to be variable in size and structure (Gent and Dawe 2012). They can be transcribed from both strands or display strand-specific characteristics (Topp et al. 2004; May et al. 2005). Some of them are exclusively nuclear while the other form cytoplasmatic polyadenylated RNA (Vourc’h and Biamonti 2011). Increasingly, evidence suggests an impact of centromeric transcripts on development, cell differentiation, and response to environmental stimuli.

Pericentromeric major satDNA in mice is highly transcribed during embryogenesis, and transcripts are responsible for reorganization of pericentromeric satDNA into chromocenters. Disruption of these transcripts led to developmental arrest indicating their role in de novo heterochromatin formation and proper developmental progression (Probst et al. 2010). In humans, polyadenylated RNA transcripts from the pericentromeric region of the Y chromosome are involved in *trans*-splicing in the CDC2L2 kinase mRNA generating a testis-specific isoform (Jehan et al. 2007). This example illustrates specific regulation of euchromatic gene expression by pericentromeric transcripts and provides a link between satDNA transcription and cell differentiation. The overexpression of centromeric RNA transcripts may be the result of derepression of heterochromatic regions under disease or stress conditions. So, it has been proposed that the differential transcription of human pericentromeric satellite III in response to heat-shock stress might be a consequence of inhibition or saturation of the RNAi machinery in the pericentromeric region (Jolly et al. 2004). BRCA1-deficient tumor cells show defective pericentromeric heterochromatin formation which leads to the disruption of gene silencing and activation of the pericentromeric alpha-satDNA transcription (Zhu et al. 2011). Derepression of satDNA transcription has also been detected in many human epithelial tumors, but it is not clear whether satDNA transcription causes or is a consequence of genomic instability and tumorigenesis (Ting et al. 2011).

In addition to the analysis of pericentromeric regions, an ever-growing number of studies on the centromere core domain demonstrates the transcription of repetitive sequences from this region and suggests a contribution of these transcripts to centromere/kinetochore assembly and maintenance (Gent and Dawe 2012). The single-stranded centromeric alpha-satellite RNA and the centromere protein CENP-C associate and facilitate nucleoprotein assembly (CENP-C, innercentromere protein INCENP, and INCENP-interacting protein survivin) at the human mitotic centromere (Wong et al. 2007). Inhibition of RNA polymerase II activity, which results in depletion of alpha-satellite RNA in mitotic human cells, reduces CENP-C binding at the kinetochore and leads to chromosome missegregation (Chan et al. 2012). Similarly, Minor satDNA transcripts from the mouse centromere are integral components of the CENP-A chromatin fraction and associate with proteins of the chromosomal passenger complex Aurora B, survivin, and INCENP. In addition to a role in mediating interactions between protein components in the centromere/kinetochore complex, it has also been evidenced that Minor satellite RNA controls the enzymatic function of the Aurora A kinase (Ferri et al. 2009). In addition to centromeric satDNA transcripts, transcripts derived from retrotransposons were also shown to be essential components of the centromere core. For example, in maize, single-stranded non-siRNAs (40–200 nt) transcribed from centromeric CentC satDNA and CRM retrotransposon are tightly bound to CENH3 (Topp et al. 2004). Similarly, RNA transcripts of the LINE-1 retrotransposon were found to bind CENP-A chromatin in Mardel (10) 10q25 neocentromere (Chueh et al. 2009). RNAi-mediated knockdown of the LINE transcripts led to a significant reduction in the mitotic stability of the neocentromere suggesting that retrotransposable elements are a critical epigenetic determinant of the neocentromere. A novel class of small RNAs encompassing contiguous satellites and retroviruses located at the centromere core and likely produced through the activity of retroviral LTR promoters was discovered in a marsupial (Carone et al. 2009). In-depth analysis discovered that hypermorphic expression of these retroelement-encoded small RNAs is critical for the maintenance and assembly of CENP-A in the marsupial centromere (Carone et al. 2013).

## Conclusions

Although being essential for the proper distribution of genetic material in eukaryotic cells, the centromere still continues to intrigue in the complexity of its structure and rapid evolution of its building components. Advances in methodological approaches and high-throughput analyses in the last two decades fostered the rapid accumulation of centromere-related datasets in different model organisms, giving access to information about DNA, RNA, proteins, and their epigenetic modifications. However, the complex networks of interactions among them as well as the details of functional features and roles of particular components are still far from being well understood. Epigenetic determinants are recognized as major identifiers of centromeres in higher eukaryotes, while the functional contribution of DNA remains obscure and seriously questioned because of the ability of the centromere to be formed and to persist on extremely diverse sequences. Recent studies of genomic and functional datasets based on combined sequencing data and established CENH3-associated DNA sequences revealed a more detailed insight into genomic architecture of centromeres. In spite of the diversity of DNA sequences, the preferred forms populating functional centromeres appear to be tandem repetitions of satDNAs and/or mobile elements. Only a subset of centromere-located DNA sequences or their variants is predominantly CENH3-associated, indicating the importance of their linear composition. An increasing number of reports that evidence organisms with dually organized centromeres (repeat-rich and repeat-free) opens up the possibility that the dynamics of centromere formation is much higher than previously thought, and also highlights stable functioning of centromeres established on different sequence types within a single organism. It can be hypothesized that the repetitive DNA environment has the potential to preserve stability of the functional centromere, and at the same time, to provide a reservoir of new functional sequences. This creates a platform which allows rapid changes in centromere identity and as a consequence can directly stimulate reproductive isolation. Several reasons for this continuous rapid change can be considered, such as specificities of evolution of satDNAs, targeted integration of TEs into the epigenetically marked centromeric environment, and coevolution of DNA sequences and CENH3 proteins. The complexity of the DNA sequence and functional relationships in centromeres becomes even more perplexing as a growing number of recent reports indicate roles for centromere DNA transcripts in centromere structure and function. Recent efforts have begun to decipher the rules in sequential patterns of centromeric DNA sequences and their functional interactions in different centromere types which will ultimately lead to a novel integrated view on the centromere genomics.

## References

[CR1] Alkan C, Cardone MF, Catacchio CR (2011). Genome-wide characterization of centromeric satellites from multiple mammalian genomes. Genome Res.

[CR2] Alonso A, Hasson D, Cheung F, Warburton PE (2010). A paucity of heterochromatin at functional human neocentromeres. Epigenetics Chromatin.

[CR3] Bachmann L, Raab M, Sperlich D (1989). Satellite DNA and speciation—a species-specific satellite DNA of *Drosophila guanche*. J Zool Syst Evol Res.

[CR4] Black BE, Bassett EA (2008). The histone variant CENP-A and centromere specification. Curr Opin Cell Biol.

[CR5] Blower MD, Sullivan BA, Karpen GH (2002). Conserved organization of centromeric chromatin in flies and humans. Dev Cell.

[CR6] Buchwitz BJ, Ahmad K, Moore LL, Roth MB, Henikoff S (1999). A histone-H3-like protein in *C. elegans*. Nature.

[CR7] Burrack LS, Berman J (2012). Neocentromeres and epigenetically inherited features of centromeres. Chromosome Res.

[CR8] Canapa A, Barucca M, Cerioni PN, Olmo E (2000). A satellite DNA containing CENP-B box-like motifs is present in the antarctic scallop *Adamussium colbecki*. Gene.

[CR9] Carbone L, Nergadze SG, Magnani E (2006). Evolutionary movement of centromeres in horse, donkey, and zebra. Genomics.

[CR10] Carone DM, Longo MS, Ferreri GC, Hall L, Harris M, Shook N, Bulazel KV, Carone BR, Obergfell C, O'Neill MJ (2009). A new class of retroviral and satellite encoded small RNAs emanates from mammalian centromeres. Chromosoma.

[CR11] Carone DM, Zhang C, Hall LE, Obergfell C, Carone BR, O’Neill MJ, O’Neill RJ (2013). Hypermorphic expression of centromeric retroelement-encoded small RNAs impairs CENP-A loading. Chromosome Res.

[CR12] Chan FL, Wong LH (2012). Transcription in the maintenance of centromere chromatin identity. Nucleic Acids Res.

[CR13] Chan FL, Marshall OJ, Saffery R, Kim BW, Earle E, Choo KH, Wong LH (2012). Active transcription and essential role of RNA polymerase II at the centromere during mitosis. Proc Natl Acad Sci U S A.

[CR14] Cheng Z, Dong F, Langdon T, Ouyang S, Buell CR, Gu M, Blattner FR, Jiang J (2002). Functional rice centromeres are marked by a satellite repeat and a centromere-specific retrotransposon. Plant Cell.

[CR15] Chueh AC, Northrop EL, Brettingham-Moore KH, Choo KH, Wong LH (2009). LINE retrotransposon RNA is an essential structural and functional epigenetic component of a core neocentromeric chromatin. PLoS Genet.

[CR16] Copenhaver GP, Nickel K, Kuromori T, Benito MI, Kaul S, Lin X, Bevan M, Murphy G, Harris B, Parnell LD, McCombie WR, Martienssen RA, Marra M, Preuss D (1999). Genetic definition and sequence analysis of Arabidopsis centromeres. Science.

[CR17] Dawe RK, Henikoff S (2006). Centromeres put epigenetics in the driver's seat. Trends Biochem Sci.

[CR18] De la Herran R, Fontana F, Lanfredi M, Congiu L, Leis M, Rossi R, Ruiz Rejón C, Ruiz Rejón M, Garrido-Ramos MA (2001). Slow rates of evolution and sequence homogenization in an ancient satellite DNA family of sturgeons. Mol Biol Evol.

[CR19] Dernburg AF (2001). Here, there, and everywhere: kinetochore function on holocentric chromosomes. J Cell Biol.

[CR20] Dong F, Miller JT, Jackson SA, Wang G-L, Ronald PC, Jiang J (1998). Rice (*Oryza sativa*) centromeric regions consist of complex DNA. Proc Natl Acad Sci U S A.

[CR21] Dover GA (1986). Molecular drive in multigene families: how biological novelties arise, spread and are assimilated. Trends Genet.

[CR22] Durajlija-Žinić S, Ugarković Đ, Cornudella L, Plohl M (2000). A novel interspersed type of organization of satellite DNAs in *Tribolium madens* heterochromatin. Chromosome Res.

[CR23] Eichler EE (1999). Repetitive conundrums of centromere structure and function. Hum Mol Genet.

[CR24] Ferree PM, Barbash DA (2009). Species-specific heterochromatin prevents mitotic chromosome segregation to cause hybrid lethality in *Drosophila*. PLoS Biol.

[CR25] Ferri F, Bouzinba-Segard H, Velasco G, Hubé F, Francastel C (2009). Non-coding murine centromeric transcripts associate with and potentiate Aurora B kinase. Nucleic Acids Res.

[CR26] Fishman L, Saunders A (2008). Centromere-associated female meiotic drive entails male fitness costs in monkeyflowers. Science.

[CR27] Fry K, Salser W (1977). Nucleotide sequences of HS- alpha satellite DNA from kangaroo rat *Dipodomys ordii* and characterization of similar sequences in other rodents. Cell.

[CR28] Fukagawa T, Nogami M, Yoshikawa M, Ikeno M, Okazaki T, Takami Y, Nakayama T, Oshimura M (2004). Dicer is essential for formation of the heterochromatin structure in vertebrate cells. Nat Cell Biol.

[CR29] Gaffney PM, Pierce JC, Mackinley AG, Titchen DA, Glenn WK (2003). Pearl, a novel family of putative transposable elements in bivalve mollusks. J Mol Evol.

[CR30] Gassmann R, Rechtsteiner A, Yuen KW (2012). An inverse relationship to germline transcription defines centromeric chromatin in *C. elegans*. Nature.

[CR31] Gent JI, Dawe RK (2012). RNA as a structural and regulatory component of the centromere. Annu Rev Genet.

[CR32] Gindullis F, Desel C, Galasso I, Schmidt T (2001). The large-scale organization of the centromeric region in Beta species. Genome Res.

[CR33] Gong Z, Wu Y, Koblízková A (2012). Repeatless and repeat-based centromeres in potato: implications for centromere evolution. Plant Cell.

[CR34] Grishok A, Tabara H, Mello CC (2000). Genetic requirements for inheritance of RNAi in *C. elegans*. Science.

[CR35] Guenatri M, Bailly D, Maison C, Almouzni G (2004). Mouse centric and pericentric satellite repeats form distinct functional heterochromatin. J Cell Biol.

[CR36] Haizel T, Lim YK, Leitch AR, Moore G (2005). Molecular analysis of holocentric centromeres of *Luzula species*. Cytogenet Genome Res.

[CR37] Hall IM, Noma K, Grewal SI (2003). RNA interference machinery regulates chromosome dynamics during mitosis and meiosis in fission yeast. Proc Natl Acad Sci U S A.

[CR38] Han F, Lamb JC, Birchler JA (2006). High frequency of centromere inactivation resulting in stable dicentric chromosomes of maize. Proc Natl Acad Sci U S A.

[CR39] Han Y, Zhang Z, Liu C, Liu J, Huang S, Jiang J, Jin W (2009). Centromere repositioning in cucurbit species: implication of the genomic impact from centromere activation and inactivation. Proc Natl Acad Sci U S A.

[CR40] Hasson D, Alonso A, Cheung F, Tepperberg JH, Papenhausen PR, Engelen JJ, Warburton PE (2011). Formation of novel CENP-A domains on tandem repetitive DNA and across chromosome breakpoints on human chromosome 8q21 neocentromeres. Chromosoma.

[CR41] Hayden KE, Willard HF (2012). Composition and organization of active centromere sequences in complex genomes. BMC Genomics.

[CR42] Hayden KE, Strome ED, Merrett SL, Lee HR, Rudd MK, Willard HF (2013). Sequences associated with centromere competency in the human genome. Mol Cell Biol.

[CR43] Heckmann S, Schroeder-Reiter E, Kumke K, Ma L, Nagaki K, Murata M, Wanner G, Houben A (2011). Holocentric chromosomes of *Luzula elegans* are characterized by a longitudinal centromere groove, chromosome bending, and a terminal nucleolus organizer region. Cytogenet Genome Res.

[CR44] Heckmann S, Macas J, Kumke K, Fuchs J, Schubert V, Ma L, Novák P, Neumann P, Taudien S, Platzer M, Houben A (2013). The holocentric species *Luzula elegans* shows interplay between centromere and large-scale genome organization. Plant J.

[CR45] Henikoff S, Ahmad K, Malik HS (2001). The centromere paradox: Stable inheritance with rapidly evolving DNA. Science.

[CR46] Hillier LW, Miller RD, Baird SE, Chinwalla A, Fulton LA, Koboldt DC, Waterston RH (2007). Comparison of *C. elegans* and *C. briggsae* genome sequences reveals extensive conservation of chromosome organization and synteny. PLoS Biol.

[CR47] Horvath JE, Gulden CL, Vallente RU, Eichler MY, Ventura M, McPherson JD, Graves TA, Wilson RK, Schwartz S, Rocchi M, Eichler EE (2005). Punctuated duplication seeding events during the evolution of human chromosome 2p11. Genome Res.

[CR48] Hosouchi T, Kumekawa N, Tsuruoka H, Kotani H (2002). Physical map-based sizes of the centromeric regions of *Arabidopsis thaliana* chromosomes 1, 2, and 3. DNA Res.

[CR49] Hyman AA, Sorger PK (1995). Structure and function of kinetochores in budding yeast. Annu Rev Cell Dev Biol.

[CR50] Jehan Z, Vallinayagam S, Tiwari S, Pradhan S, Singh L, Suresh A, Reddy HM, Ahuja YR, Jesudasan RA (2007). Novel noncoding RNA from human Y distal heterochromatic block (Yq12) generates testis-specific chimeric CDC2L2. Genome Res.

[CR51] Jolly C, Metz A, Govin J, Vigneron M, Turner BM, Khochbin S, Vourc'h C (2004). Stress-induced transcription of satellite III repeats. J Cell Biol.

[CR52] Kalitsis P, Choo KH (2012). The evolutionary life cycle of the resilient centromere. Chromosoma.

[CR53] Kanellopoulou C, Muljo SA, Kung AL, Ganesan S, Drapkin R, Jenuwein T, Livingston DM, Rajewsky K (2005). Dicer-deficient mouse embryonic stem cells are defective in differentiation and centromeric silencing. Genes Dev.

[CR54] Kapitonov VV, Holmquist GP, Jurka J (1998). L1 repeat is a basic unit of heterochromatin satellites in cetaceans. Mol Biol Evol.

[CR55] Kasai F, Garcia C, Arruga MV, Ferguson-Smith MA (2003). Chromosome homology between chicken (*Gallus gallus domesticus*) and the red-legged partridge (*Alectoris rufa*); evidence of the occurrence of a neocentromere during evolution. Cytogenet Genome Res.

[CR56] Kato A, Lamb JC, Birchler JA (2004). Chromosome painting using repetitive DNA sequences as probes for somatic chromosome identification in maize. Proc Natl Acad Sci U S A.

[CR57] Kazazian HH (2004). Mobile elements: Drivers of genome evolution. Science.

[CR58] Kipling D, Warburton PE (1997). Centromeres, CENP-B and Tigger too. Trends Genet.

[CR59] Koo DH, Han F, Birchler JA, Jiang J (2011). Distinct DNA methylation patterns associated with active and inactive centromeres of the maize B chromosome. Genome Res.

[CR60] Kumekawa N, Hosuchi T, Tsuruoka H, Kotani H (2000). The size and sequence organization of the centromeric region of *Arabidopsis thaliana* chromosome 5. DNA Res.

[CR61] Kuznetsova I, Podgornaya O, Ferguson-Smith MA (2006). High-resolution organization of mouse centromeric and pericentromeric DNA. Cytogenet Genome Res.

[CR62] Lam AL, Boivin CD, Bonney CF, Rudd MK, Sullivan BA (2006). Human centromeric chromatin is a dynamic chromosomal domain that can spread over noncentromeric DNA. Proc Natl Acad Sci U S A.

[CR63] Lamb JC, Meyer JM, Birchler JA (2007). A hemicentric inversion in the maize line knobless Tama flint created two sites of centromeric elements and moved the kinetochore-forming region. Chromosoma.

[CR64] Lee HR, Zhang W, Langdon T, Jin W, Yan H, Cheng Z, Jiang J (2005). Chromatin immunoprecipitation cloning reveals rapid evolutionary patterns of centromeric DNA in Oryza species. Proc Natl Acad Sci U S A.

[CR65] Li B, Choulet F, Heng Y, Hao W, Paux E, Liu Z, Yue W, Jin W, Feuillet C, Zhang X (2013). Wheat centromeric retrotransposons: the new ones take a major role in centromeric structure. Plant J.

[CR66] Ma J, Jackson SA (2006). Retrotransposon accumulation and satellite amplification mediated by segmental duplication facilitate centromere expansion in rice. Genome Res.

[CR67] Ma J, Wing RA, Bennetzen JL, Jackson SA (2007). Plant centromere organization: a dynamic structure with conserved functions. Trends Genet.

[CR68] Macas J, Koblízková A, Navrátilová A, Neumann P (2009). Hypervariable 3' UTR region of plant LTR-retrotransposons as a source of novel satellite repeats. Gene.

[CR69] Macas J, Neumann P, Novák P, Jiang J (2010). Global sequence characterization of rice centromeric satellite based on oligomer frequency analysis in large-scale sequencing data. Bioinformatics.

[CR70] Mahtani MM, Willard HF (1998). Physical and genetic mapping of the human X chromosome centromere: repression of recombination. Genome Res.

[CR71] Malik HS, Henikoff S (2001). Adaptive evolution of Cid, a centromere-specific histone in Drosophila. Genetics.

[CR72] Malik HS, Henikoff S (2009). Major evolutionary transitions in centromere complexity. Cell.

[CR73] Maloney KA, Sullivan LL, Matheny JE, Strome ED, Merrett SL, Ferris A, Sullivan BA (2012). Functional epialleles at an endogenous human centromere. Proc Natl Acad Sci U S A.

[CR74] Marshall OJ, Chueh AC, Wong LH, Choo KH (2008). Neocentromeres: new insights into centromere structure, disease development, and karyotype evolution. Am J Hum Genet.

[CR75] Masabanda JS, Burt DW, O'Brien PC (2004). Molecular cytogenetic definition of the chicken genome: the first complete avian karyotype. Genetics.

[CR76] Masumoto H, Nakano M, Ohzeki J (2004). The role of CENP-B and alpha-satellite DNA: de novo assembly and epigenetic maintenance of human centromeres. Chromosome Res.

[CR77] May BP, Lippman ZB, Fang Y, Spector DL, Martienssen RA (2005). Differential regulation of strand-specific transcripts from *Arabidopsis* centromeric satellite repeats. PLoS Genet.

[CR78] Melters DP, Bradnam KR, Young HA (2013). Comparative analysis of tandem repeats from hundreds of species reveals unique insights into centromere evolution. Genome Biol.

[CR79] Meštrović N, Plohl M, Mravinac B, Ugarković Đ (1998). Evolution of satellite DNAs from the genus *Palorus*—Experimental evidence for the "library" hypothesis. Mol Biol Evol.

[CR80] Meštrović N, Pavlek M, Car A, Castagnone-Sereno P, Abad P, Plohl M (2013). Conserved DNA motifs, including the CENP-B box-like, are possible promoters of satellite DNA array rearrangements in nematodes. PLoS One.

[CR81] Mola LM, Papeschi AG (2006). Holokinetic chromosomes at a glance. BAG J Basic Appl Genet.

[CR82] Mravinac B, Plohl M (2010). Parallelism in evolution of highly repetitive DNAs in sibling species. Mol Biol Evol.

[CR83] Mravinac B, Plohl M, Meštrović N, Ugarković Đ (2002). Sequence of PRAT satellite DNA "frozen" in some Coleopteran species. J Mol Evol.

[CR84] Mravinac B, Ugarković Đ, Franjević D, Plohl M (2005). Long inversely oriented subunits form a complex monomer of *Tribolium brevicornis* satellite DNA. J Mol Evol.

[CR85] Mravinac B, Sullivan LL, Reeves JW, Yan CM, Kopf KS, Farr CJ, Schueler MG, Sullivan BA (2009). Histone modifications within the human X centromere region. PLoS One.

[CR86] Murchison EP, Partridge JF, Tam OH, Cheloufi S, Hannon GJ (2005). Characterization of Dicer-deficient murine embryonic stem cells. Proc Natl Acad Sci U S A.

[CR87] Nagaki K, Talbert PB, Zhong CX, Dawe RK, Henikoff S, Jiang J (2003). Chromatin immunoprecipitation reveals that the 180-bp satellite repeat is the key functional DNA element of *Arabidopsis thaliana* centromeres. Genetics.

[CR88] Nagaki K, Cheng Z, Ouyang S, Talbert PB, Kim M, Jones KM, Henikoff S, Buell CR, Jiang J (2004). Sequencing of a rice centromere uncovers active genes. Nat Genet.

[CR89] Nagaki K, Kashihara K, Murata M (2005). Visualization of diffuse centromeres with centromere-specific histone H3 in the holocentric plant *Luzula nivea*. Plant Cell.

[CR90] Neumann P, Navrátilová A, Koblížková A, Kejnovský E, Hřibová E, Hobza R, Widmer A, Doležel J, Macas J (2011). Plant centromeric retrotransposons: a structural and cytogenetic perspective. Mob DNA.

[CR91] Neumann P, Navrátilová A, Schroeder-Reiter E, Koblížková A, Steinbauerová V, Chocholová E, Novák P, Wanner G, Macas J (2012). Stretching the rules: monocentric chromosomes with multiple centromere domains. PLoS Genet.

[CR92] Ohta T, Dover GA (1984). The cohesive population genetics of molecular drive. Genetics.

[CR93] Ohzeki J, Nakano M, Okada T, Masumoto H (2002). CENP-B box is required for de novo centromere chromatin assembly on human alphoid DNA. J Cell Biol.

[CR94] Okamoto Y, Nakano M, Ohzeki J, Larionov V, Masumoto H (2007). A minimal CENP-A core is required for nucleation and maintenance of a functional human centromere. EMBO J.

[CR95] Pal-Bhadra M, Leibovitch BA, Gandhi SG, Rao M, Bhadra U, Birchler JA, Elgin SC (2004). Heterochromatic silencing and HP1 localization in *Drosophila* are dependent on the RNAi machinery. Science.

[CR96] Palomeque T, Lorite P (2008). Satellite DNA in insects: a review. Heredity.

[CR97] Pimpinelli S, Berloco M, Fanti L, Dimitri P, Bonaccorsi S, Marchetti E, Caizzi R, Caggese C, Gatti M (1995). Transposable elements are stable structural components of *Drosophila melanogaster* heterochromatin. Proc Natl Acad Sci U S A.

[CR98] Piras FM, Nergadze SG, Magnani E, Bertoni L, Attolini C, Khoriauli L, Raimondi E, Giulotto E (2010). Uncoupling of satellite DNA and centromeric function in the genus Equus. PLoS Genet.

[CR99] Plohl M, Luchetti A, Meštrović N, Mantovani B (2008) Satellite DNAs between selfishness and functionality: structure, genomics and evolution of tandem repeats in centromeric (hetero)chromatin. Gene 409:72–82 10.1016/j.gene.2007.11.013 18182173

[CR100] Plohl M, Meštrović N, Mravinac B, Garrido-Ramos MA (2012). Satellite DNA evolution. Repetitive DNA, Genome dynamics.

[CR101] Pluta AF, Mackay AM, Ainsztein AM, Goldberg IG, Earnshaw WC (1995). The centromere: hub of chromosomal activities. Science.

[CR102] Probst AV, Okamoto I, Casanova M, El Marjou F, Le Baccon P, Almouzni G (2010). A strand-specific burst in transcription of pericentric satellites is required for chromocenter formation and early mouse development. Dev Cell.

[CR103] Reyes-Turcu FE, Zhang K, Zofall M, Chen E, Grewal SIS (2011). Defects in RNA quality control factors reveal RNAi-independent nucleation of heterochromatin. Nat Struct Mol Biol.

[CR104] Rocchi M, Archidiacono N, Schempp W, Capozzi O, Stanyon R (2012). Centromere repositioning in mammals. Heredity.

[CR105] Rudd MK, Willard H (2004). Analysis of the centromeric regions of the human genome assembly. Trends Genet.

[CR106] Rudd MK, Wray GA, Willard HF (2006). The evolutionary dynamics of alpha-satellite. Genome Res.

[CR107] Sato H, Masuda F, Takayama Y, Takahashi K, Saitoh S (2012). Epigenetic inactivation and subsequent heterochromatinization of a centromere stabilize dicentric chromosomes. Curr Biol.

[CR108] Schueler MG, Higgins AW, Rudd MK, Gustashaw K, Willard HF (2001). Genomic and genetic definition of a functional human centromere. Science.

[CR109] Shang WH, Hori T, Toyoda A, Kato J, Popendorf K, Sakakibara Y, Fujiyama A, Fukagawa T (2010). Chickens possess centromeres with both extended tandem repeats and short non-tandem-repetitive sequences. Genome Res.

[CR110] Sharma A, Wolfgruber TK, Presting GG (2013). Tandem repeats derived from centromeric retrotransposons. BMC Genomics.

[CR111] Smith GP (1976). Evolution of repeated DNA sequences by unequal crossover. Science.

[CR112] Smith CD, Shu S, Mungall CJ, Karpen GH (2007). The Release 5.1 annotation of *Drosophila melanogaster* heterochromatin. Science.

[CR113] Spence JM, Critcher R, Ebersole TA, Valdivia MM, Earnshaw WC, Fukagawa T, Farr CJ (2002). Co-localization of centromere activity, proteins and topoisomerase II within a subdomain of the major human X alpha-satellite array. EMBO J.

[CR114] Stimpson KM, Song IY, Jauch A, Holtgreve-Grez H, Hayden KE, Bridger JM, Sullivan BA (2010). Telomere disruption results in non-random formation of de novo dicentric chromosomes involving acrocentric human chromosomes. PLoS Genet.

[CR115] Strachan T, Webb D, Dover GA (1985). Transition stages of molecular drive in multiple-copy DNA families in *Drosophila*. EMBO J.

[CR116] Sullivan BA, Willard HF (1998). Stable dicentric X chromosomes with two functional centromeres. Nat Genet.

[CR117] Sullivan BA, Karpen GH (2004). Centromeric chromatin exhibits a histone modification pattern that is distinct from both euchromatin and heterochromatin. Nat Struct Mol Biol.

[CR118] Sullivan LL, Boivin CD, Mravinac B, Song IY, Sullivan BA (2011). Genomic size of CENP-A domain is proportional to total alpha satellite array size at human centromeres and expands in cancer cells. Chromosome Res.

[CR119] Sun X, Wahlstrom J, Karpen G (1997). Molecular structure of a functional *Drosophila* centromere. Cell.

[CR120] Sun X, Le HD, Wahlstrom JM, Karpen GH (2003). Sequence analysis of a functional Drosophila centromere. Genome Res.

[CR121] Talbert PB, Henikoff S (2010). Centromeres convert but don’t cross. PLoS Biol.

[CR122] Talbert PB, Masuelli R, Tyagi AP, Comai L, Henikoff S (2002). Centromeric localization and adaptive evolution of an *Arabidopsis* histone H3 variant. Plant Cell.

[CR123] Talbert PB, Bryson TD, Henikoff S (2004). Adaptive evolution of centromere proteins in plants and animals. J Biol.

[CR124] Thompson SL, Bakhoum SF, Compton DA (2010). Mechanisms of chromosomal instability. Curr Biol.

[CR125] Ting DT, Lipson D, Paul S (2011). Aberrant overexpression of satellite repeats in pancreatic and other epithelial cancers. Science.

[CR126] Tollis M, Boissinot S, Garrido-Ramos MA (2012). The evolutionary dynamics of transposable elements in eukaryote genomes. Repetitive DNA, Genome dynamics.

[CR127] Topp CN, Zhong CX, Dawe RK (2004). Centromere-encoded RNAs are integral components of the maize kinetochore. Proc Natl Acad Sci U S A.

[CR128] Tsukahara S, Kawabe A, Kobayashi A, Ito T, Aizu T, Shin-i T, Toyoda A, Fujiyama A, Tarutani Y, Kakutani T (2012). Centromere-targeted de novo integrations of an LTR retrotransposon of Arabidopsis lyrata. Genes Dev.

[CR129] Ventura M, Antonacci F, Cardone MF, Stanyon R, D'Addabbo P, Cellamare A, Sprague LJ, Eichler EE, Archidiacono N, Rocchi M (2007). Evolutionary formation of new centromeres in macaque. Science.

[CR130] Volpe TA, Kidner C, Hall IM, Teng G, Grewal SI, Martienssen RA (2002). Regulation of heterochromatic silencing and histone H3 lysine-9 methylation by RNAi. Science.

[CR131] Vourc'h C, Biamonti G (2011). Transcription of Satellite DNAs in Mammals. Prog Mol Subcell Biol.

[CR132] Waye JS, Willard HF (1989). Human beta satellite DNA: genomic organization and sequence definition of a class of highly repetitive tandem DNA. Proc Natl Acad Sci U S A.

[CR133] Willard HF, Waye JS (1987). Chromosome-specific subsets of human alpha satellite DNA: analysis of sequence divergence within and between chromosomal subsets and evidence for an ancestral pentameric repeat. J Mol Evol.

[CR134] Wolfgruber TK, Sharma A, Schneider KL (2009). Maize centromere structure and evolution: sequence analysis of centromeres 2 and 5 reveals dynamic Loci shaped primarily by retrotransposons. PLoS Genet.

[CR135] Wong LH, Brettingham-Moore KH, Chan L (2007). Centromere RNA is a key component for the assembly of nucleoproteins at the nucleolus and centromere. Genome Res.

[CR136] Wu J, Yamagata H, Hayashi-Tsugane M (2004). Composition and structure of the centromeric region of rice chromosome 8. Plant Cell.

[CR137] Zhang W, Friebe B, Gill BS, Jiang J (2010). Centromere inactivation and epigenetic modifications of a plant chromosome with three functional centromeres. Chromosoma.

[CR138] Zhong CX, Marshall JB, Topp C, Mroczek R, Kato A, Nagaki K, Birchler JA, Jiang JM, Dawe RK (2002). Centromeric retroelements and satellites interact with maize kinetochore protein CENH3. Plant Cell.

[CR139] Zhu Q, Pao GM, Huynh AM, Suh H, Tonnu N, Nederlof PM, Gage FH, Verma IM (2011). BRCA1 tumour suppression occurs via heterochromatin-mediated silencing. Nature.

[CR140] Zilberman D, Cao X, Jacobsen SE (2003). ARGONAUTE4 control of locus-specific siRNA accumulation and DNA and histone methylation. Science.

